# Co-expression of Cas9 and single-guided RNAs in *Escherichia coli* streamlines production of Cas9 ribonucleoproteins

**DOI:** 10.1038/s42003-019-0402-x

**Published:** 2019-05-03

**Authors:** Jie Qiao, Wenqiang Li, Siyu Lin, Wenli Sun, Lixin Ma, Yi Liu

**Affiliations:** 1 State Key Laboratory of Biocatalysis and Enzyme Engineering, School of Life Sciences, Hubei University, Wuhan, 430062 Hubei China; 2 Hubei Collaborative Innovation Center for Green Transformation of Bio-resources, Wuhan, 430062 Hubei China; 3 Hubei Key Laboratory of Industrial Biotechnology, School of Life Sciences, Wuhan, 430062 Hubei China

**Keywords:** Biological techniques, Biotechnology, CRISPR-Cas9 genome editing

## Abstract

CRISPR/Cas9 ribonucleoprotein (RNP) complexes are promising biological tools with diverse biomedical applications. However, to date there are no efficient methods that can produce these proteins at large scales and low cost. Here, we present a streamlined method for direct production of Cas9 RNPs from *Escherichia coli* by co-expression of Cas9 and the target-specific single-guided RNAs. Harnessing an ultrahigh-affinity CL7/Im7 purification system recently developed we achieve one-step purification of the self-assembling CRISPR/Cas RNPs, including the commonly used Cas9 and Cas12a, within half a day and with a ~fourfold higher yield than incumbent methods. The prepared Cas RNPs show remarkable stability in the absence of RNase inhibitors, as well as profound gene-editing efficiency in vitro and in vivo. Our method is convenient, cost-effective, and can be used to prepare other CRISPR/Cas RNPs.

## Abstract (web summary)

Jie Qiaoa, Wenqiang Lia, Siyu Lina et al. develop a cost-efficient method to prepare stable and efficient CRISPR/Cas ribonucleoprotein complexes. Using a one-step purification system with an ultra-high-affinity CL7/Im7 column they successfully harvested high yields of functional Cas9 and target-specific single guided RNAs co-expressed in *Escherichia coli*.

## Introduction

Clustered regularly interspaced short palindromic repeats (CRISPR)/CRISPR-associated protein (Cas) genome-editing systems, including the widely used nucleases *Streptococcus pyogenes* Cas9 (SpCas9)^1,2^ and *Francisella novicida* Cas12a (previously named FnCpf1)^3,4^, have been extensively applied to biomedical area with great promises to revolutionize the treatment of genetic diseases^5,6^. To date, genetic therapy with adeno-associated viruses is still the most advanced approach for delivering CRISPR systems in vivo^7^; however, this methodology has fundamental shortcomings such as the risk of carcinogenesis, limited insertion size^8^, and immune responses. In comparison to the viral methods, plenty of researches recently demonstrated that direct delivery of CRISPR/Cas ribonucleoproteins (RNPs) for genome editing in cells and animals has obvious advantages^9–11^, such as reduced off-target effects, low toxicity, high-editing efficiency, etc. Therefore, several biopharmaceutical companies are now paying emphasis on developing Cas RNP-based gene therapeutic medicines.

The current strategy for generating Cas RNPs is relatively time consuming and expensive^12–14^, because the recombinant Cas enzymes and the single-guided RNAs (sgRNAs) were individually produced, followed by assembly of them in vitro in certain ratios. Typically, the Cas enzymes are expressed and purified from *E. coli*, whereas the sgRNAs are constructed either by in vitro transcription or by chemical synthesis. Therefore, the development of a straightforward and cost-effective method to produce Cas RNPs remains a challenge.

Recently, we achieved co-expression of Cas9 enzymes and the associated guide RNAs in *E. coli* to prepare self-assembling Cas9 RNPs^15^. To purify these Cas9 RNPs, we harnessed a first Ni-NTA affinity purification and a following gel filtration step, resulting in a yield of ~10 mg Cas9 RNPs from 1 L LB culture medium. In this work, we utilize a newly developed ultrahigh-affinity CL7/Im7 purification system^16^ to realize one-step purification of CRISPR/Cas RNPs, including the widely used Cas9 and Cas12a, with a fourfold higher yield than incumbent methods. Meanwhile, the purification time course is largely reduced from 2 or 3 days to half a day. In this system, the CL7 tag that engineered from the *E. coli* Colicin E7 DNase (CE7) retains the ultrahigh-binding affinity (*K*_D_ ≈ 10^−14^–10^−17^ M) with its inhibitor Immunity protein 7 (Im7)^16^, which is much higher than the His-trap approach (*K*_D_ ≈ 10^−8^–10^−9^ M). Importantly, the Im7 affinity column can be conveniently prepared by ourselves, or directly purchased from TriAltus bioscience (Birmingham, AL, USA). The Cas RNPs prepared by the method show remarkable stability in the absence of RNase inhibitors, as well as profound gene editing efficiency in vitro and in vivo. Collectively, we establish a valid platform to simply produce CRISPR/Cas RNPs at low cost and short time.

## Results

### Direct expression of CL7-tagged Cas9 RNPs from *E. coli*

To obtain fully formed Cas9 RNPs, the current strategy is to roughly mix Cas9 enzymes and the associated sgRNAs in vitro^17^. The major shortcoming of this methodology is that the sgRNAs are often susceptible to enzymatic degradation during the process by ubiquitous RNases in the environment. Therefore, it is challenging to load high-quality sgRNAs in a sufficient amount to Cas9. We recently established a co-expression method to directly prepare Cas9 RNPs in *E. coli*^15^. By the approach, the newly synthesized Cas9 enzymes and the transcribed sgRNAs were spontaneously self-assembled within *E. coli* cells, forming matured Cas9/sgRNA complexes. We found that such kind of self-assembling Cas9 RNPs are very stable which maintain full activity at −20 °C for up to 9 months in the absence of RNase inhibitors. The methodology still has limitations yet, two of which are the relatively low yield and the long purification time.

**Fig. 1:**
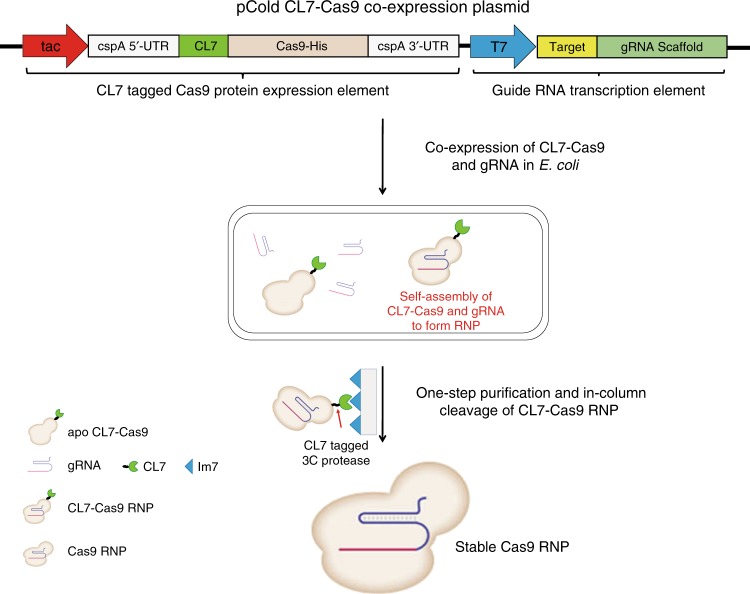
An engineered cold-shock expression vector was harnessed to achieve co-expression of CL7–Cas9 and sgRNA in *E. coli*. The CL7–Cas9 and sgRNAs were spontaneously self-assembled within *E. coli* cells to form CL7–Cas9 RNPs. The pure Cas9 RNPs with high stability were prepared by one-step purification and in-column cleavage of CL7 tags using a CL7-tagged HRV 3C protease

To increase the yields of Cas9 RNPs, here we introduced a CL7 tag in the N-terminus of original Cas9^16^. The CL7 tag can be easily removed by human rhinovirus (HRV) 3C proteinase recognized cleavage at 16 °C for 3 h^18^. In addition, to prevent contamination of the 3C proteinase in the final sample, an engineered CL7-tagged HRV 3C proteinase was used. The scheme of expression plasmid termed pCold CL7–Cas9 was shown in Fig. 1. The CL7 is a catalytically inactive variant of Colicin E7 (CE7) DNase with a low *K*_D_ (∼10^−14^–10^−17^ M) toward its binding partner Im7^19^. This CL7/Im7 system has recently been reported to facilitate purification of diverse proteins as well as to enhance their production^16^. According to the design, the sgRNA molecules were abundantly transcribed in *E. coli* when adding IPTG, while the CL7–Cas9 fusion proteins were simultaneously expressed within *E. coli* too. The yield of Cas9 RNPs was increased to ~40 mg/L when using LB culture medium, which is fourfold higher than incumbent methods. Moreover, we applied the method to produce Cas12a RNPs, and also resulted in a much higher yield (~30 mg/L) than the current method which uses maltose binding protein as the fusion tag^3^. All the gene sequences and plasmid maps are shown in Supplementary Figs. 1–5. The NCBI gene identification for the proteins used in this work are: *S. pyogenes* Cas9, Gene ID: 901176; *F. novicida* Cas12a, Gene ID: 2827873; *E. coli* Colicin E7 DNase (CE7), Gene ID: 20467019. Interestingly, we found that the CL7–Cas9 RNP has a similar endonuclease activity (Supplementary Fig. 6) to Cas9 RNP, indicating that the CL7-tagged variant can be alternatively used for genome editing.

### One-step purification of Cas RNPs by CL7/Im7 ultrahigh-affinity system

**Fig. 2:**
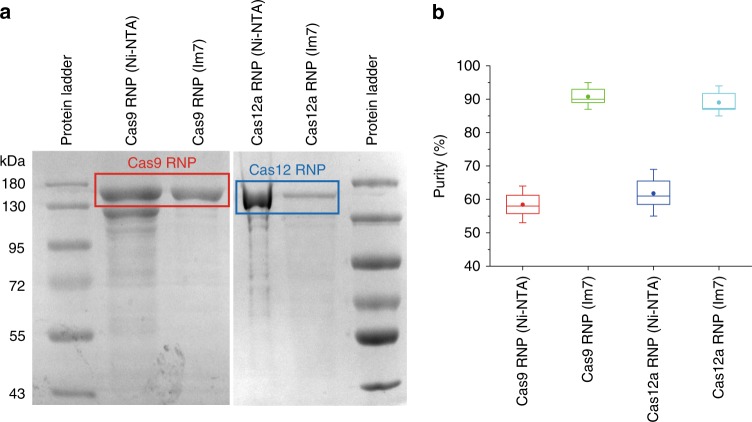
**a** A total of 12% SDS–PAGE of Cas9 RNP (10 μg, red square) and Cas12a RNP (5 μg, blue square) purified by Ni-NTA affinity column or by Im7 column. The original uncropped gels were shown in Supplementary Fig. 7. **b** The target Cas RNPs’ purity was validated from three individual batches of purification by gray scanning analysis (ImageJ) of the SDS–PAGE. Data are shown as the mean ± SD

To purify Cas9 RNPs, we previously harnessed a Ni-NTA affinity purification followed by a gel filtration step using the HiLoad 26/60 Superdex 200 column (GE, USA)^15^. During the multistep purification, a large number of Cas9 enzymes might be lost. In addition, two or more days are needed to prepare Cas RNPs. Herein, the introduction of an ultrahigh-affinity CL7/Im7 system^16^ helped us achieving one-step purification of Cas RNPs within half a day (Supplementary Table 1, see Supplementary Information for comparative details). Compared with the Cas9 RNPs purified by Ni-NTA affinity column, the purity of Cas9 RNPs obtained by Im7 column was increased from ~58% to ~89% based on the gray scanning analysis (Fig. 2). The various bands visible on the gel for Cas RNPs purified by Ni-NTA were proteins from *E. coli* itself. The purity of target Cas RNPs can be improved by gradient elution using different concentrations of imidazole follow by a second purification step using gel filtration. Importantly, reproducible results were observed for preparation of Cas9 RNPs with different sgRNAs (Supplementary Fig. 7). In addition, there were no batch-to-batch variations regarding production of the same Cas9 RNPs. Notably, the Im7 affinity column was simply prepared by ourselves through coupling the recombinant Im7 enzymes to agarose beads, and can be repeatedly regenerated without losing its binding affinity to the CL7 affinity tag^16^. So, the high expense of Ni-NTA agarose (~2000 USD for 100 mL from Qiagen, USA) can be saved, making our method very cost-effective. Alternatively, the Im7 ligated agarose beads can be purchased from TriAltus bioscience (Birmingham, AL, USA).

The CRISPR/Cas RNPs prepared by incumbent methods^17^ are often unstable which might loss activities within weeks due to the digestion of sgRNAs, even in the presence of RNase inhibitors. By our method, none of RNase inhibitors are yet required in the whole purification and storage processes. The Cas9 RNPs were found very stable which can be stored at −20 °C for 9 months without activity changes. To explain the observations, we proposed that the sgRNAs transcribed in vivo within *E. coli* could somehow tightly bind to the nascent Cas9, helping Cas9 fold into a stable conformation which protects sgRNAs from nuclease-mediated degradation.

### Cleavage of vectors by Cas9 RNPs as artificial restriction endonucleases

Restriction enzymes are essential genetic tools for recombinant DNA technology that have revolutionized modern biological research since the early 1970s^20^. So far, there are over 250 commercially available restriction endonucleases for routine uses in thousands of laboratories around the world^21^. Most of them only recognize short-DNA sequences (typically ~6 or 8 bp), which limits their applications in particular use such as seamless DNA cloning. Recently, Wang et al. have successfully employed Cas9 enzymes as artificial restriction enzymes (AREs) that combined with Gibson assembly to accomplish seamless DNA cloning^22^. Yet, it is still difficult to prepare AREs in a sufficient amount at low cost and short time, challenging their wide applications instead of restriction enzymes. To our knowledge, our aforementioned method addressed this issue for the first time, enabling timely and inexpensive production of Cas RNPs.

**Fig. 3:**
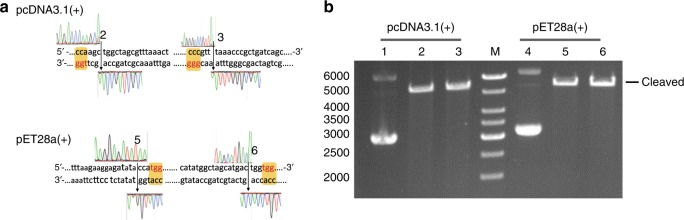
**a** Cleavage of plasmids including pCDNA3.1(+) and pET28a(+) at MCS by two Cas9 RNPs. **b** The results of Cas9 RNPs cleavage detected by 0.8% agarose page. The cleavage sites were indicated with arrows and numbered in Fig. 3a corresponding to the lanes in Fig. 3b

As known, Cas9 recognizes a ~20 bp target sequence with a required downstream NRG (where R = G or A) PAM and induces a site-specific double strand break^23,24^. We searched all PAM sites at the multiple clone sites in popularly used vectors, such as pET28a (+) and pcDNA3.1 (+) (Fig. 3). Taking pcDNA3.1(+), for example, it has 12 restriction enzyme sites and 19 PAM sites in the same region. Even better, we can simply introduce new PAM sites at any desired location in the vectors. It is, therefore, more than enough to generate a sufficient amount of Cas9 RNPs instead of restriction enzymes. More importantly, the multiple PAM sites can facilitate researchers to choose specific sites for molecular cloning in certain situations that the restriction enzymes were forbidden. For instance, we cannot use a restriction enzyme if its recognizing sequence exists both in cloning DNA and vector. Nevertheless, we do not have to worry about the issue when using Cas9 RNPs, because there is a very low probability that the same 20 bp recognizing sequence resides both in cloning DNA and vector. At present, we have prepared a series of Cas9 RNPs as AREs instead of the commercial restriction enzymes for molecular cloning in our laboratory.

### In vitro nuclease cleavage and in vivo genome editing by Cas RNPs

**Fig. 4:**
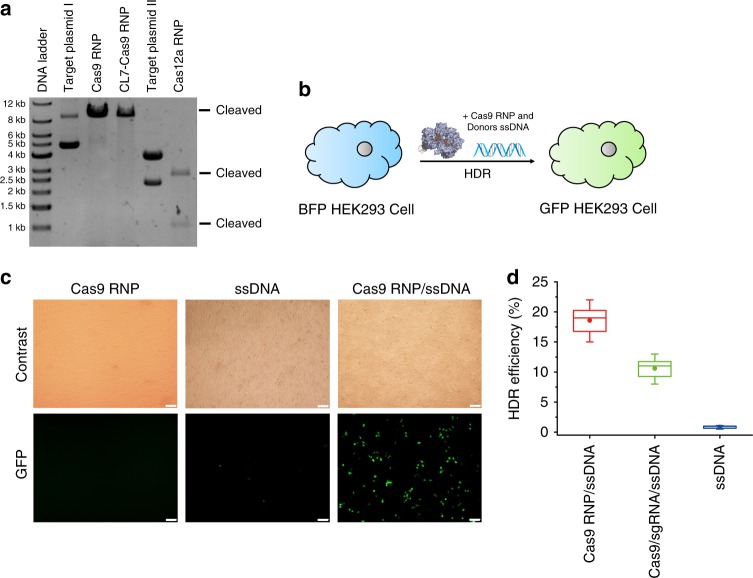
The endonuclease activity assays of purified Cas RNPs. **a** In vitro cleavage on the target plasmid I (single cleavage site) by Cas9 RNPs or CL7–Cas9 RNPs, as well as on the target plasmid II (two cleavage sites) by Cas12a RNPs. **b** Delivery of Cas9 RNPs and donor ssDNA in BFP-HEK293 cells can induce HDR-mediated genome editing which can convert them into GFP-HEK 293 cells. **c** The bright-filed and fluorescent images of BFP-HEK293 cells after delivery of Cas9 RNPs (left), donor ssDNA only (middle), and Cas9 RNPs together with ssDNA donor (right) by lipofectamine CRISPRMAX in 48 h later. Scale bars: 100 μM. **d** The HDR efficiency was determined by GFP expression due to BFP editing according to the flow cytometry data (Supplementary Figure 8), including the prepared Cas9 RNPs in this work with ssDNA donor (red), ssDNA donor only (blue), and Cas9 enzyme). Data are shown as the mean ± SD

To determine the in vitro nuclease activity of Cas RNPs, we found that 300 ng plasmids were fully cleaved in less than 30 min at 37 °C when adding 200 ng of Cas RNPs (Fig. 4a), including Cas9 RNP, Cas12a RNP, and CL7–Cas9 RNP. The nuclease activity of produced Cas RNPs is comparable to the commercial CRISPR/Cas enzymes, which indicates that our method can be industrially applicable to produce CRISPR/Cas RNPs.

To illustrate the efficiency of in vivo homology dependent repair (HDR)-mediated genome editing by Cas RNPs, we constructed an engineered blue fluorescent protein (BFP)-expressing HEK293 cell line as the reporter system^25^. When correct genome repairing occurred after co-delivery of Cas9 RNPs and a 70 nt ssDNA donor (see the sequence in Supplementary Information) into cells by lipofectamine CRISPRMAX (Fig. 4b), the BFP-HEK293 cells would be converted to green fluorescent protein (GFP)-expressing cells. The HDR efficiencies were estimated from the flow cytometry data (Supplementary Fig. 8) and shown in Fig. 4d. The HDR efficiency of Cas9 RNPs produced by our method is ~19%, which is 1.8 times higher than incumbent methods (~11%). In contrast, none of HDR efficiency was observed when delivering Cas9 RNPs individually and ~1% of HDR frequencies were obtained when transferring ssDNA donor only. Collectively, these results indicate that the prepared Cas9 RNPs have profound gene-editing efficiency in vitro and in vivo.

## Discussion

Several works have been proven that injection of Cas9 RNPs into tissues and organs is a promising strategy for the treatment of genetic diseases^5,26^, however, the most challenge remaining is that the RNPs must be stable enough to survive degradation in organisms^27^. Limited protein lifetime will require delivery of higher doses of Cas9 RNPs into the patient, resulting in poor target gene editing. Conversely, delivering the CRISPR/Cas RNPs with improved thermostability showed an increased lifetime in human plasma^27^. Besides, CRISPR-based screening of genomic DNA has recently enabled studying both genetic and noncoding gene regulatory elements, but construction of high-throughput screening platforms using CRISPR/Cas RNP-based libraries have not yet been established, largely due to the production of such RNPs is relatively time-consuming and expensive by incumbent methods. Here, we report a streamlined method to timely produce and purify CRISPR/Cas RNPs at low cost from *E. coli*. More importantly, we demonstrated that the prepared Cas9 RNPs have enhanced stability which can be effectively used for HDR-mediated genome editing in mammalian cells. In future, we could apply the platform to produce other gRNA-loaded CRISPR/Cas RNPs.

## Methods

### Design and construction of the co-expression plasmids

We ordered genes including CL7, Cas9, and Cas12a with optimizations using *E. coli* biased codons from Sangon Biotech (Shanghai, China). We cloned a CL7 tag in the N-terminus of Cas9 or Cas12a with insertion of a HRV 3C proteinase recognition site between them (Fig. 1). In addition, we preserved the C-terminal 6 x His tag for purification of CL7-tagged fusion enzymes. An engineered cold-shock vector pCold I (Takara, Japan) was used for construction of the expression plasmids, in which the original cspA promoter was replaced with a tac promoter as well as the f1 ori was replaced with a T7 promoter. NCBI gene identifications for the proteins used in this work are: *S. pyogenes* Cas9, Gene ID: 901176; *F. novicida* Cas12a, Gene ID: 2827873; *E. coli* Colicin E7 DNase (CE7), Gene ID: 20467019. All the gene sequences are shown in the Supplementary Figs. 1–4. In addition, the plasmids contained a sgRNA transcription element that can be easily substituted by any wanted sgRNA via *Sal* I digestion. Herein, we generated two expression plasmids termed pCold CL7–Cas9 and pCold CL7–Cas12a, respectively (Supplementary Fig. 5). We deposited both plasmids along with maps and sequences in Addgene.

### Preparation of Im7-ligated agarose beads

Im7 gene was synthesized by Sangon Biotech (Shanghai, China) and cloned into a pET23a(+) expression vector. The Im7 proteins were over-expressed in *E. coli* BL21(DE3) cells and purified according to the protocol^16^. Then, we cross-linked them covalently to 1 mM iodoacetyl agarose beads from Sigma-Aldrich. After cleavage and elution of the target proteins, such as CL7–Cas9 RNP or CL7–Cas12a RNP, with a CL7-tagged HRV 3C proteinase, the cleaved CL7 tags and CL7-3C proteinases bound to the Im7 agarose beads can be readily removed by treatment with 6 M Guanidine hydrochloride (Sinopharm Group Co. Ltd., China). The column was then regenerated via in-column Im7 refolding^16^. Recently, the Im7 ligated agarose beads are commercially available by TriAltus bioscience (Birmingham, AL, USA).

### Purification of Cas RNPs by Im7 column in a single step

The co-expression plasmids aforementioned were transformed into *E. coli* Rosetta (DE)3 cells and then cultured at 37 °C till OD_600_ reached 0.8. Then, we added 0.5 mM IPTG and turned down the temperature to 16 °C for expression of Cas RNPs. The cells were harvested and lysed in lysis buffer (20 mM Tris-HCl, pH 7.4, 100 mM NaCl). The CL7-tagged Cas9 RNPs or Cas12a RNPs were loaded onto the Im7 column. After washing two cycles of washing buffer I (20 mM Tris-HCl, pH 7.4, 300 mM NaCl), the CL7-3C protease was added to the column for in-column cleavage of CL7 tags at 16 °C for three hours. Next, Cas RNPs were eluted with washing buffer II (20 mM Tris-HCl, pH 7.4, 500 mM NaCl). Finally, the Cas RNPs were concentrated and stored at −80 °C in the storage buffer (20 mM Tris-HCl, pH 7.4, 500 mM NaCl, 20% glycerin).

### In vitro endonuclease activity assay

For the in vitro endonuclease activity assay, the purified Cas RNPs were directly applied to digest the plasmids containing target dsDNA sequences. The digestion was typically carried out in a 10 μL volume of reaction mixture, composed of 1 μL 10× buffer 3.1 (NEB), 200 ng Cas9 or Cas12a RNPs, as well as 300 ng plasmids, at 37 °C for 30 min followed by termination of the reaction at 80 °C for 10 min. The cleaved DNA fragmentation was evaluated by 2% agarose gel electrophoresis (Supplementary Fig. 6).

### In vivo genome-editing assay

The original HEK293 cell line was from Biovector NTCC Inc (Beijing, China). For illustrating efficiency of the in vivo HDR-mediated genome editing by Cas RNPs, we constructed a BFP-expressing HEK293 reporter cell line according to the protocol^25^. To edit BFP to GFP, the Cas9 RNPs were delivered together with a 70 nt donor ssDNA by lipofectamine CRISPRMAX^28^ (Thermol Fisher, USA). The HDR efficiency was calculated from the flow cytometry data using the GFP cell counts/total cell counts (Supplementary Fig. 8).

### Reporting summary

Further information on experimental design is available in the Nature Research Reporting Summary linked to this article.

## Supplementary information

Supplementary Information

Reporting Summary

## Data Availability

The authors declare that all relevant data supporting the findings of this study are available within the article and its Supplementary information files. The plasmids and DNA sequences were deposited in Addgene under the following Addgene Plasmid IDs: 124890 (pCold CL7-Cas9) and 124891 (pCold CL7-Cas12a).
