# Author Correction: Co-expression of Cas9 and single-guided RNAs in *Escherichia coli* streamlines production of Cas9 ribonucleoproteins

**DOI:** 10.1038/s42003-019-0695-9

**Published:** 2019-11-27

**Authors:** Jie Qiao, Wenqiang Li, Siyu Lin, Wenli Sun, Lixin Ma, Yi Liu

**Affiliations:** 1 State Key Laboratory of Biocatalysis and Enzyme Engineering, School of Life Sciences, Hubei University, Wuhan, 430062 Hubei China; 2 Hubei Collaborative Innovation Center for Green Transformation of Bio-resources, Wuhan, 430062 Hubei China; 3 Hubei Key Laboratory of Industrial Biotechnology, School of Life Sciences, Wuhan, 430062 Hubei China

Correction to: *Communications Biology* 10.1038/s42003-019-0402-x, published online 03 May 2019.

In the original version of this Article, the Addgene plasmid identifier in the Data Availability statement was incorrectly given as 76583. The correct identifiers are 124890 for plasmid pCold CL7-Cas9 and 124891 for plasmid pCold CL7-Cas12a. The error has been corrected in the HTML and PDF versions of the Article.

