# Supplementary material for: Co-expression of Cas9 and single-guided RNAs in Escherichia coli streamlines production of Cas9 ribonucleoproteins
Source: Commun Biol. 2019 May 3;2:161. doi: 10.1038/s42003-019-0402-x (PMC6499778; doi:10.1038/s42003-019-0402-x)
Supplement: Supplementary file 1 — Supplementary Information [file 42003_2019_402_MOESM1_ESM.pdf]

Supplementary Information

Supplementary Figure 1

5’gtggtcggggtagcggctgaagcactgcacgccgtacgtcagggtggtcacgagggtgggccagggc
acg-3’

Supplementary Figure 1. The sequence of ssDNA donor for converting BFP-expressing HEK293 cells to GFP-expressing HEK293 cells.

Supplementary Figure 2

5’atgagcaaaagcaatgaaccgggtaaagcaaccggtgaaggtaaaccggttaataacaaatggctg
aacaatgccggtaaagatctgggtagtccggttccggatcgtattgcaaataaactgcgtgataaagaattc
gagagcttcgatgattttcgtgaaaccttttgggaagaagttagcaaagatcctgaactgagcaaacagttta
gccgcaataacaatgatcgtatgaaagttggtaaagcaccgaaaacacgtacccaggatgttagcggtaa
acgtacctcatttgaactgaatcatcagaaaccgattgaacagaatggtggcgtttatgatatggataacatt
agcgttgttaccccgaaacgcaacattgatattgaaggcggtggtggtggttctttggaggttttgttccagggt
ccaatggataagaaatactcaataggcttagatatcggcacaaatagcgtcggatgggcggtgatcactga
tgaatataaggttccgtctaaaaagttcaaggttctgggaaatacagaccgccacagtatcaaaaaaaatct
tataggggctcttttatttgacagtggagagacagcggaagcgactcgtctcaaacggacagctcgtagaa
ggtatacacgtcggaagaatcgtatttgttatctacaggagattttttcaaatgagatggcgaaagtagatgat
agtttctttcatcgacttgaagagtcttttttggtggaagaagacaagaagcatgaacgtcatcctatttttggaa
atatagtagatgaagttgcttatcatgagaaatatccaactatctatcatctgcgaaaaaaattggtagattcta
ctgataaagcggatttgcgcttaatctatttggccttagcgcatatgattaagtttcgtggtcatttttttgattgagg
gagatttaaatcctgataatagtgatgtggacaaactatttatccagttggtacaaacctacaatcaattatttg
aagaaaaccctattaacgcaagtggagtagatgctaaagcgattctttctgcacgattgagtaaatcaagac
gattagaaaatctcattgctcagctccccggtgagaagaaaaatggcttatttgggaatctcattgctttgtcatt
gggtttgacccctaattttaaatcaaattttgatttggcagaagatgctaaattacagctttcaaaagatacttac
gatgatgatttagataatttattggcgcaaattggagatcaatatgctgatttgtttttggcagctaagaatttatc
agatgctattttactttcagatatcctaagagtaaatactgaaataactaaggctcccctatcagcttcaatgatt
aaacgctacgatgaacatcatcaagacttgactcttttaaaagctttagttcgacaacaacttccagaaaagt
ataaagaaatcttttttgatcaatcaaaaaacggatatgcaggttatattgatgggggagctagccaagaag
aattttataaatttatcaaaccaattttagaaaaaatggatggtactgaggaattattggtgaaactaaatcgtg
aagatttgctgcgcaagcaacggacctttgacaacggctctattccccatcaaattcacttgggtgagctgca
tgctattttgagaagacaagaagacttttatccatttttaaaagacaatcgtgagaagattgaaaaaatcttga
cttttcgcattccttattatgttggtccattggcgcgtggcaatagtcgttttgcatggatgactcggaagtctgaa
gaaacaattaccccatggaattttgaagaagttgtcgataaaggtgcttcagctcaatcatttattgaacgcat
gacaaactttgataaaaatcttccaaatgaaaaagtactaccaaaacatagtttgctttatgagtattttacggt
ttataacgaattgacaaaggtcaaatatgttactgaaggaatgcgaaaaccagcatttctttcaggtgaaca
gaagaaagccattgttgatttactcttcaaaacaaatcgaaaagtaaccgttaagcaattaaaagaagatta
tttcaaaaaaatagaatgttttgatagtgttgaaatttcaggagttgaagatagatttaatgcttcattaggtacct
accatgatttgctaaaaattattaaagataaagatttttttggataatgaagaaaatgaagatatcttagaggat
attgttttaacattgaccttatttgaagatagggagatgattgaggaaagacttaaaacatatgctcacctctttg
atgataaggtgatgaaacagcttaaacgtcgccgttatactggttggggacgtttgtctcgaaaattgattaat
ggtattagggataagcaatctggcaaaacaatattagatttttttgaaatcagatggttttgccaatcgcaattta
tgcagctgatccatgatgatagtttgacatttaaagaagacattcaaaaagcacaagtgtctggacaaggc
gatagtttacatgaacatattgcaaatttagctggtagccctgctattaaaaaaggtattttacagactgtaaaa
gttgttgatgaattggtcaaagtaatggggcggcataagccagaaaatatcgttattgaaatggcacgtgaa
aatcagacaactcaaaagggccagaaaaattcgcgagagcgtatgaaacgaatcgaagaaggtatca
aagaattaggaagtcagattcttaaagagcatcctgttgaaaatactcaattgcaaaatgaaaagctctatct
ctattatctccaaaatggaagagacatgtatgtggaccaagaattagatattaatcgtttaagtgattatgatgt
cgatcacattgttccacaaagtttccttaaagacgattcaatagacaataaggtcttaacgcgttctgataaaa
atcgtggtaaatcggataacgttccaagtgaagaagtagtcaaaaagatgaaaaactattggagacaactt

ctaaacgccaagttaatcactcaacgtaagtttgataatttaacgaaagctgaacgtggaggtttgagtgaa
cttgataaagctggttttatcaaacgccaattggttgaaactcgccaaatcactaagcatgtggcacaaatttt
ggatagtcgcatgaatactaaatacgatgaaaatgataaacttattcgagaggttaaagtgattaccttaaaa
tctaaattagtttctgacttccgaaaagatttccaattctataaagtacgtgagattaacaattaccatcatgccc
atgatgcgtatctaaatgccgtcgttggaactgctttgattaagaaatatccaaaacttgaatcggagtttgtct
atggtgattataaagtttatgatgttcgtaaaatgattgctaagtctgagcaagaaataggcaaagcaaccgc
aaaatatttcttttactctaatatcatgaacttcttcaaaacagaaattacacttgcaaatggagagattcgcaa
acgccctctaatcgaaactaatggggaaactggagaaattgtctgggataaagggcgagattttgccacag
tgcgcaaagtattgtccatgccccaagtcaatattgtcaagaaaacagaagtacagacaggcggattctcc
aaggagtcaattttaccaaaaagaaattcggacaagcttattgctcgtaaaaaagactgggatccaaaaa
aatatggtggttttgatagtccaacggtagcttattcagtcctagtggttgctaaggtggaaaaagggaaatcg
aagaagttaaaatccgttaaagagttactagggatcacaattatggaaagaagttcctttgaaaaaaatccg
attgactttttagaagctaaaggatataaggaagttaaaaaagacttaatcattaaactacctaaatatagtctt
tttgagttagaaaacggtcgtaaacggatgctggctagtgccggagaattacaaaaaggaaatgagctgg
ctctgccaagcaaatatgtgaatttttatatttagctagtcattatgaaaagttgaagggtagtccagaagata
acgaacaaaaacaattgtttgtggagcagcataagcattatttagatgagattattgagcaaatcagtgaattt
tctaagcgtgttattttagcagatgccaatttagataaagttcttagtgcatataacaaacatagagacaaacc
aatacgtgaacaagcagaaaatattattcatttatttacgttgacgaatcttggagctcccgctgcttttaaatatt
ttgatacaacaattgatcgtaaacgatatacgtctacaaaagaagttttagatgccactcttatccatcaatcc
atcactggtctttatgaaacacgcattgatttgagtcagctaggaggtgac-3’

Black: CL7 protein

Green: 3C proteinase recognition site

Yellow: Cas9 protein

Supplementary Figure 2. The gene sequence encoding CL7-Cas9.

Supplementary Figure 3

5’atgagcaaaagcaatgaaccgggtaaagcaaccggtgaaggtaaaccggttaataacaaatggctg
aacaatgccggtaaagatctgggtagtccggttccggatcgtattgcaaataaactgcgtgataaagaattc
gagagcttcgatgattttcgtgaaaccttttgggaagaagttagcaaagatcctgaactgagcaaacagttta
gccgcaataacaatgatcgtatgaaagttggtaaagcaccgaaaacacgtacccaggatgttagcggtaa
acgtacctcatttgaactgaatcatcagaaaccgattgaacagaatggtggcgtttatgatatggataacatt
agcgttgttaccccgaaacgcaacattgatattgaaggcggtggtggtggttctttggaggttttgttccagggt
ccaatgtcaatttatcaagaatttgtgaacaaatatagcctgagcaaaaccctgcgttttgaactgattccgca
gggtaaaaccctggaaaacattaaagcacgtggtctgattctggatgatgaaaaacgtgccaaagactac
aaaaaagccaaacaaatcatcgataaataccaccagttcttcatcgaagaaattctgagcagcgtttgcatt
agcgaagatctgctgcagaattattccgacgtttatttcaaactgaaaaaaagcgacgatgataacctgcag
aaagatttcaaaagcgccaaagataccatcaaaaaacaaattagcgagtatatcaaagacagcgagaa
attcaaaaacctgttcaaccagaatctgatcgatgccaaaaaaggtcaagaaagcgatctgatcctgtggc
tgaaacagagcaaagataatggcatcgaactgtttaaagccaacagcgatattaccgatattgatgaagc
actggaaatcatcaaaagctttaaaggttggaccacctactttaaaggctttcacgaaaatcgcaaaaacgt
gtatagcagcaatgatattccgaccagcattatctatcgcatcgttgatgataatctgcctaaatttctggaaaa
taaagccaaatatgaaagcctgaaagacaaagcaccggaagcaattaactatgagcagatcaaaaaa
gatctggccgaagaactgacctttgacattgattacaaaaccagcgaagttaaccagcgtgtttttagcctgg
atgaagtttttgaaattgccaacttcaacaactacctgaatcagagcggtatcaccaaattcaataccattatc
ggtggcaaattcgtgaatggcgaaaataccaaacgcaaaggcatcaacgaatacattaatctgtatagcc
agcagattaacgataaaacgctgaaaaaatacaaaatgagcgtgctgttcaaacaaattctgtcagatacc
gaaagcaaaagcttcgtgattgacaaactggaagatgatagtgatgttgttaccaccatgcagagcttttatg
aacaaatcgcagcgtttaaaaccgtggaagagaaatccattaaagaaaccctgagcctgctgtttgatgat
ctgaaagcacagaaactggacctgtccaaaatctacttcaaaaacgataaatccctgaccgatctgagcc
agcaggttttcgatgattatagcgttattggcaccgcagttctggaatatatcacacagcagattgcaccgaa
aaatctggataatccgagcaaaaaagaacaagagctgatcgccaaaaaaaccgagaaagcgaaatat
ctgagcctggaaacaattaaactggccctggaagaatttaacaaacaccgcgacattgataaacagtgcc
gctttgaagaaatcctggcaaattttgcagcaatcccgatgatctttgatgaaattgcgcagaataaagataa
cctggcacagatcagcatcaaatatcagaatcagggaaaaaaagacctgctgcaagcaagtgccgaag
atgatgttaaagcgattaaagatctgctggatcagaccaataacctgctgcataaactgaaaatctttcacatt
agccagagcgaggataaagcgaacattctggataaagatgagcacttctatctggtgtttgaagagtgttatt
ttgagctggcaaatattgtgccgctgtataacaaaatccgcaactatattacccagaaaccgtatagcgacg
aaaaattcaaactgaactttgagaatagcaccctggccaatggttgggataaaaacaaagaaccggata
ataccgccatcctgttcattaaagatgataaatactatctgggcgtgatgaacaaaaaaaacaacaaaatct
tcgacgataaagccatcaaagagaataaaggcgaaggttacaaaaaaatcgtgtacaaactgctgcctg
gtgcgaataaaatgctgccgaaagtgtttttttagcgccaaatccatcaaattctataacccgagcgaagatat
tctgcgtattcgtaatcatagcacccataccaaaaatggtagtccgcagaaaggctatgaaaaattcgagtt
caacattgaggattgccgcaaattcatcgacttctacaaacagtccattagcaaacatccggaatggaaag
actttggttttcgttttagcgatacccagcgctataacagcattgatgaattttatcgcgaagtggaaaaccagg
gctataaactgacatttgaaaacatcagcgagagctatattgatagcgttgtgaatcagggtaaactgtacct
gtttcagatctataacaaagactttagcgcctatagcaaaggtcgtccgaatctgcataccctgtattggaaa
gcactgttcgatgaacgtaatctgcaggatgttgtctacaaactgaatggtgaagcagaactgttttatcgcaa
acagagtatcccgaaaaaaatcacccatccggcaaaagaagcaatcgcgaacaaaaacaaagataa

cccgaaaaaagaaagcgtgttcgagtatgatctgatcaaagataaacgcttcaccgaagataaattctttttc
cattgcccgatcaccatcaactttaaaagcagcggtgcgaacaaattcaacgatgaaatcaatctgctgctg
aaagaaaaagccaacgatgttcatattctgagcattgatcgtggtgaacgtcatctggcctattacaccctgg
ttgatggtaaaggcaatattatcaaacaggacaccttcaacattatcggcaatgatcgtatgaaaaccaact
accatgataaactggcagccattgaaaaagatcgtgatagcgcacgtaaagattggaaaaaaatcaaca
acattaaagaaatgaaagaaggctacctgagccaggttgttcatgaaatcgccaaactggtgattgaatat
aatgccattgtggtgttcgaggatctgaacttcggtttcaaacgtggtcgtttcaaagttgagaaacaggtgtat
caaaaactggaaaaaatgctgatcgaaaaactgaattacctggtgttcaaagacaacgaattcgataaaa
ccggtggtgttctgcgtgcatatcagctgaccgcaccttttgaaaccttcaaaaaaatgggtaaacagaccg
gcatcatctattatgttccggcaggttttacctccaaaatttgtccggttaccggctttgttaatcagctgtatccga
aatatgagagcgttagcaaaagccaagagtttttcagcaaatttgataaaatctgctataacctggacaaag
gctactttgaattcagctttgactataaaaactttggcgataaagcagccaaaggcaaatggaccattgcaa
gctttggtagccgtctgattaactttcgtaacagcgacaaaaaccataactgggatacccgtgaagtttatcc
gaccaaagagctggaaaaactgctgaaagattacagcattgaatatggtcatggcgaatgtattaaagcc
gcaatttgtggtgagtccgacaaaaaattctttgcaaaactgaccagcgtgctgaataccattctgcagatgc
gtaatagcaaaaccggcaccgaactggattatctgattagtccggttgcagatgtgaacggcaatttttttcgat
agccgtcaggctccgaaaaatatgccgcaggatgcagatgcaaatggtgcctatcatattggcctgaaag
gtctgatgctgctgggtcgcattaaaaacaatcaagaaggcaaaaaactgaacctggtgatcaaaaacga
agagtattttgagttcgtgcagaataggaataactag-3’

Black: CL7 protein

Green: 3C proteinase recognition site

Yellow: Cas12a protein

Supplementary Figure 3. The gene sequence encoding CL7-Cas12a.

Supplementary Figure 4

5’taatacgactcactataggtgacatcaattattatacatgttttagagctagaaatagcaagttaaaataag
gctagtccgttatcaacttgaaaaagtggcaccgagtcggtgcttttttctagcataaccccttggggcctctaa
acgggtcttgaggggttttttg-3’

Black: T7 promoter

Yellow: gRNA

Cyan: T7 terminator

Supplementary Figure 4. The gene sequence for the accompany gRNA which targets BFP gene in this work.

Supplementary Figure 5

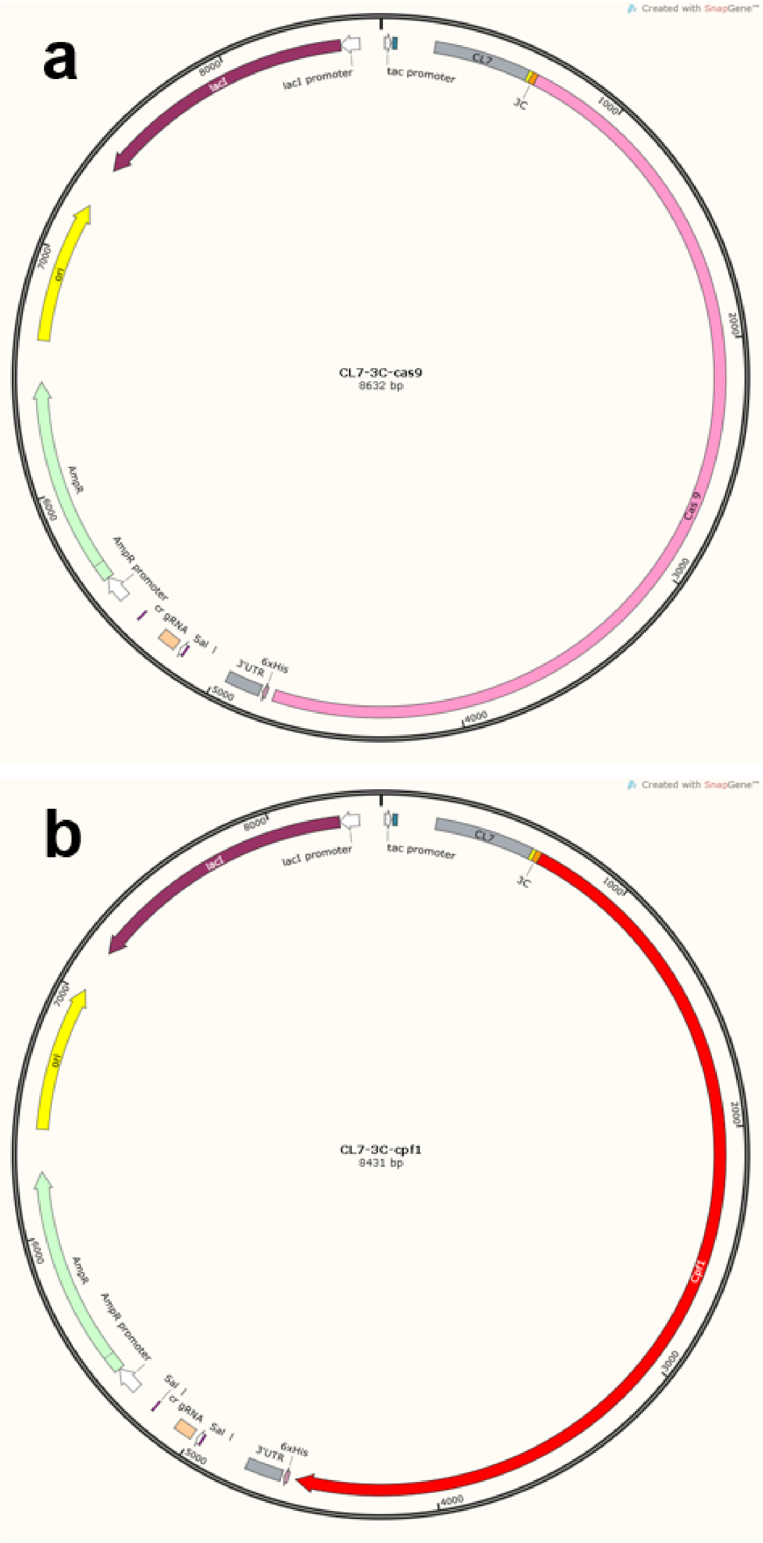

Supplementary Figure 5. (a) Plasmid map of pCold CL7-Cas9. (b) Plasmid map of pCold CL7-Cas12a.

Supplementary Figure 6

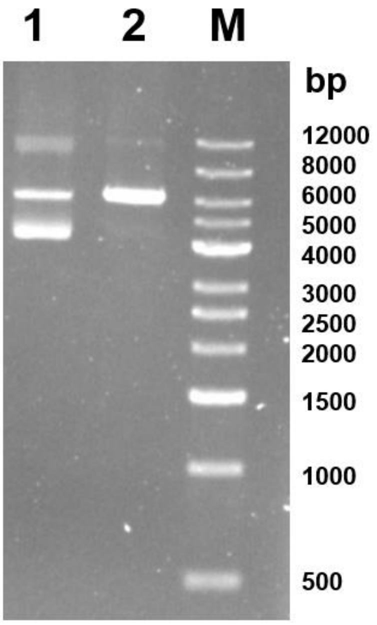

Supplementary Figure 6. Cleavage of the 300 ng target plasmids (lane 1) by 200 ng CL7-Cas9 RNPs (lane 2) produced and purified from *E. coli* cells.

Supplementary Figure 7

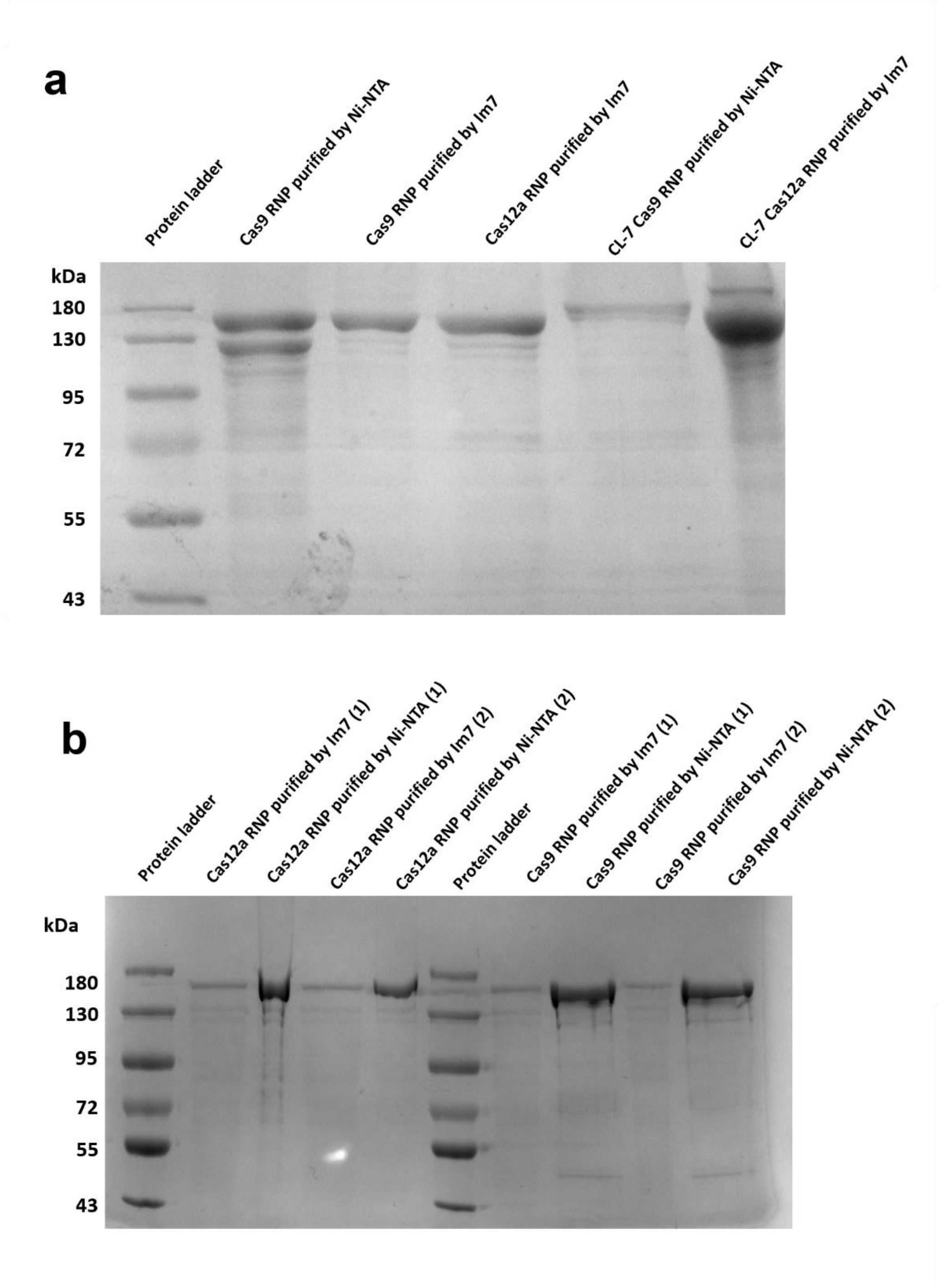

Supplementary Figure 7. (a) 12% SDS-PAGE of purified Cas RNPs by Im7 column or by Ni-NTA affinity column. (b) Two individual batches of purification of Cas12a RNP and Cas9 RNP by Im7 column or by Ni-NTA column.

Supplementary Figure 8

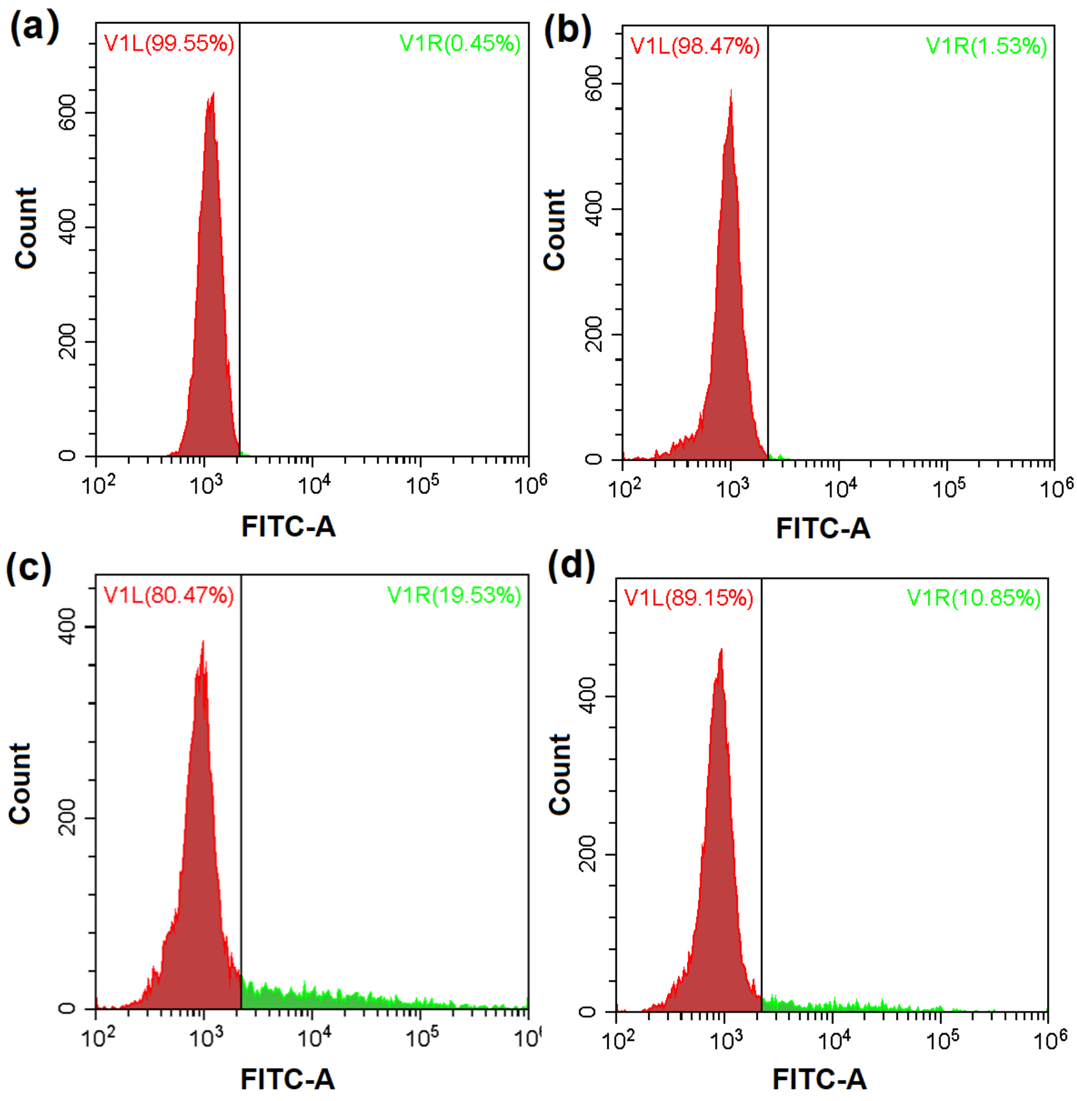

Supplementary Figure 8. Measurements of the HDR efficiency by flow cytometry. (a) HEK293 cells without transfection (control); (b) transfection of sgRNA only; (c) transfection of Cas9 RNPs (prepared by our method) and ssDNA donor by Lipofectamine CRISPRMAX; (d) transfection of Cas9 (prepared by incumbent methods), sgRNA and ssDNA donor by Lipofectamine CRISPRMAX.

Supplementary Table 1. Comparing Cas RNPs prepared by our method to the incumbent methods.

Cas RNP	Time[a] (days)	Yields[b] (mg/L)	Purity[c] (%)	Stability[d] (relative)	Cost[e] (relative)
Cas9 RNP (this work)	0.5	40	90%	Over 9 months	$
Cas9 RNP (incumbent)	0.5	10	58%	~3-4 weeks	$$$$
Cas12a RNP (this work)	2-3	30	87%	Over 9 months	$
Cas12a RNP (incumbent)	2-3	8	61%	~3-4 weeks	$$$$

a. This time means the purification time of Cas RNPs or Cas enzymes under the condition of thee *E. coli* cells were already harvested. For incumbent methods, if considering the time for chemical synthesis of sgRNA, the time for production of Cas RNPs will usually be more than 2 weeks.

b. The yields were estimated from 1 L LB culture medium.

c. The purity was estimated from SDS-page results in Figure 2. For incumbent methods, only one-step Ni-NTA purification was used herein.

d. Relative stability was validated by the *in vitro* endonuclease activity assay. The activity of Cas RNPs prepared by our method did not change after nine months when storing at -20°C in the absence of RNase inhibitors. In contrast, the activity of Cas RNPs prepared by incumbent methods was often dramatically decreased even in the presence of RNase inhibitors.

e. The cost of incumbent methods including the fees of Ni-NTA resins, HiLoad 26/60 Superdex 200 column, chemical synthesis of gRNA (or transcription kits), and RNase inhibitors, all of which can be cut down by our method. According to rough estimation, ~3/4 costs can be saved when using our method in comparison to the incumbent methods.
